# Sir Henry Morris' Decision

**Published:** 1918-06-29

**Authors:** 


### MEDICAL FEES TO FELLOW-PRACTITIONERS.

### Sir Henry Morris' Decision.

OUR readers will be interested to read the following statement relating to an action recently tried (see THE HOSPITAL, May 18, 1918, page 131), for which we are indebted to the *Lancet*.

In the recent action before Mr. Justice Bailhache in the High Court, in which Dr. F. J. Nicholls sued Mr. P. de Santi and his wife for fees incurred on account of medical attendance on the last-named, judgment was given for the defendants but, at the suggestion of the judge, the amount of any further payment to Dr. Nicholls was referred to Sir Henry Morris as arbitrator. His decision in the matter runs as follows:—

"In pursuance of the agreement reached in court before Mr. Justice Bailhache, on May 8, Dr. Nicholls, the plaintiff in the action, and Mr. de Santi, one of the defendants, attended at 8 Cavendish Square, London, W., before me on the 22nd inst. (May). Each of the parties in the presence of the other made statements and replied to questions put by me, and as a result I find the facts of the case to be as follows: A total sum of £448 4s. was paid at different times to Dr. Nicholls for medical treatment between March 7, 1912, and August 27, 1916.

"In the earlier period of the attendance the amounts paid to the plaintiff had been received by Mrs. de Santi from her step-father for this purpose.

"On June 30, 1912, Mrs. de Santi expressed her regret that Dr. Nicholls' attendance upon her must cease, as she would no longer be receiving assistance from her step-father. Dr. Nicholls, however, preferred the continuance of his services, and they were accepted, and, as a matter of fact, assistance was received by Mrs. de Santi from her step-father until September 1914, and all the money so received was paid by her to the plaintiff.

"On February 2, 1915, the plaintiff gave up attending Mrs. de Santi, but at her request he resumed attendance the next day at a fee of half a guinea for each evening visit, on the understanding, according to the statement of Mr. de Santi, that such fee was to cover the evening visit, morning visit on the following day, and all additional treatment. This fee was paid on each occasion out of moneys provided by Mr. de Santi, though Dr. Nicholls was not aware of this being the case until the meeting here. Dr. Nicholls admitted the receipt of this half-guinea fee on the occasion of each evening visit, but he stated before me that he understood such fee was only to be on account. Any misunderstanding, however, on this point—the only point of difference between the parties on the matters referred to above—is, in my opinion, immaterial, because Dr. Nicholls, in reply to a question put by me, admitted that any payments supplementary to the half-guinea fees would be made by Mrs. de Santi out of moneys received by her from an outside source, and no such moneys since the date of the arrangement made on February 3, 1915, have been received by her.

"In view of all the above facts, I do not consider that Dr. Nicholls has any moral claim to any further payment, or any ground for thinking he has not been fairly treated by his patient or her husband. I cannot think that any medical man is justified in making a demand for further payment after having received sums of money at the rate of £100 a year for professional attendance upon the family of another medical man. Indeed, during the period since the payment by Mr. de Santi himself of half-guineas commenced Dr. Nicholls has been remunerated at the rate, approximately, of £175 or £180 per annum.

"As Dr. Nicholls now knows that all these half-guinea fees were paid out of Mr. de Santi's own pocket, I am of the opinion that he should make the *amende honorable* and express regret to Mr. de Santi for the annoyance caused him by bringing an action at law against him."

